# New Working Environments: Mission Started

**DOI:** 10.1007/s00062-021-01117-y

**Published:** 2021-12-06

**Authors:** Jennifer Linn

**Affiliations:** Institute of Diagnostic and Interventional Neuroradiology, University Hospital Carl Gustav Carus, Technical University Dresden, Dresden, Germany

Considerable achievements regarding the pathomechanism and molecular pathology of a wide variety of diseases recently resulted in new, more personalized therapeutic approaches. With respect to diagnostic neuroradiology, these promising developments challenge us to broaden our diagnostic portfolio in order to enable us to detect such individual molecular features in our images.

“New working environments—mission completed?”, the motto of this year’s annual meeting of the German Society of Neuroradiology, referred partly to the efforts made in this respect. In state of the art and special focus sessions as well as scientific rounds, neuroRAD 2021 laid the emphasis on recent developments in neuro-oncology, neurodegenerative diseases, autoimmune disorders as well as on cerebrovascular diseases including vasospasm, interventional neuroradiology and cerebral small vessel diseases. Regarding the latter, latest insights in these very common diseases were comprehensively presented by Prof. Joanna Wardlaw, University of Edinburgh, in her excellent keynote lecture.

Besides these new clinical working environments, our congress motto also referred to current and future trends regarding the organization and management of our daily work. In this respect, the sessions organized by the *Junge Neuroradiologie* focused on the future of education, training and career opportunities in neuroradiology.

The inspiring keynote lecture by Sascha Friesike, Professor of Digital Innovation Design at the Berlin University of the Arts, provided a more general view on the potential impact of artificial intelligence on our field. He impressively illustrated that we should aim for digital transformation—meaning the invention of something new—rather than for pure digitalization and advised us to handle digital and technological innovations with “skeptical curiosity”. His talk emphasized the need of our active engagement in this process, which requires implementation of relevant issues in our radiological and neuroradiological curriculum.

As my personal conclusion, the new clinical and scientific developments presented at neuroRAD 2021 convincingly demonstrated that, although not yet completed, we have successfully started the mission to implement and advance very promising new working environments in the field of neuroradiology.

Prof. Dr. Jennifer Linn, Dresden

President of neuroRAD 2021,

the 56th Annual Meeting of the German Society of Neuroradiology, 6–8 October 2021

